# A newly identified novel variant in the *CSF2RA* gene in a child with pulmonary alveolar proteinosis: a case report

**DOI:** 10.1186/s13256-017-1285-4

**Published:** 2017-05-02

**Authors:** Adel S. Al-Haidary, Wadha Alotaibi, Sami A. Alhaider, Suhail Al-Saleh

**Affiliations:** 1 Department of Pediatrics, King Fahad Medical City, P.O. Box 59046, Riyadh, 11525 Saudi Arabia; 2 Department of Pediatrics, King Faisal Specialist Hospital and Research Centre, Riyadh, Saudi Arabia; 3 The Hospital for Sick Children, 555 University Avenue, Toronto, M5G 1X8 ON Canada

**Keywords:** Pulmonary alveolar proteinosis, *CSF2RA*, Whole lung lavage, Pediatrics, Diffuse lung disease, case report

## Abstract

**Background:**

The congenital form of pulmonary alveolar proteinosis due to colony stimulating factor 2 receptor alpha gene mutations is a rare disease with only a few cases reported worldwide. In this study we report a new case of pulmonary alveolar proteinosis with a novel variant in colony stimulating factor 2 receptor alpha gene.

**Case presentation:**

A 5-year-old Saudi boy presented with a history of progressive dyspnea over 6 months; he was diagnosed as having pulmonary alveolar proteinosis. A molecular study revealed a novel variation in colony stimulating factor 2 receptor alpha gene. His clinical condition showed significant improvement after whole lung lavage.

**Conclusions:**

This case has the typical presentation of congenital pulmonary alveolar proteinosis due to colony stimulating factor 2 receptor alpha defect with a novel variant in this gene likely to be pathogenic.

## Background

Pulmonary alveolar proteinosis (PAP) is a respiratory disease characterized by the accumulation of surfactant within alveoli leading to respiratory distress and hypoxemic respiratory failure in severe cases [1, 2]. PAP is a very rare disease with an estimated prevalence of 0.1 per 100,000 individuals; in more than 90% of cases, PAP is an autoimmune disease, less commonly it is of the congenital type [3, 4]. The congenital form of PAP is caused by colony stimulating factor 2 receptor alpha (*CSF2RA*) gene mutation with only a few cases reported worldwide [5–7]. We report a new pediatric case of PAP with the typical presentation of congenital PAP due to *CSF2RA* gene defect with a novel variant in this gene and good response to therapeutic whole lung lavage (WLL).

## Case presentation

A 5-year-old Saudi boy presented with exertional dyspnea that progressed over 6 months to dyspnea and hypoxemia at rest. He is the first-born of his parents who were first cousins; he has a healthy 3.5-year-old sister. His past medical and familial history were uneventful. He had a mild cough that sometimes contained whitish sputum. His weight was decreasing. He had no history of fever, chest pain, environmental exposure to causative agents, or drug use. His development was appropriate for his age. A clinical examination revealed respiratory distress (respiratory rate was 55 per minute, the oxygen saturation was 95% on 5 L/minute face mask oxygen) with mild degree of clubbing of fingers. His body weight and height were less than third centile: body weight 9.85 kg and height 97.5 cm. A chest examination revealed decreased air movements bilaterally with crackles.

**Fig. 1:**
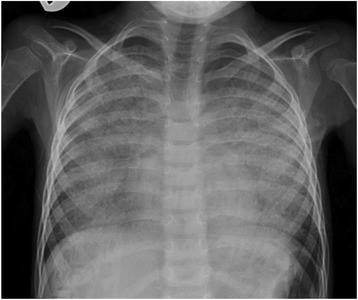
Chest X-ray showed widespread bilateral air-space disease

**Fig. 2:**
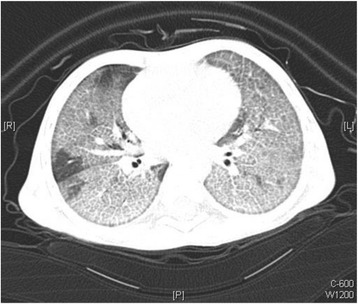
Computed tomographic scan of the chest showed ground-glass opacification and interlobular septal thickening (crazy paving pattern)

His complete blood count, urea and electrolytes, immune work up, human immunodeficiency virology (HIV) serology, thyroid function test, and echocardiography were all within normal range. A chest X-ray showed widespread bilateral air-space disease (Fig. 1). Metabolic screening (tandem mass spectrometry, urine gas chromatography-mass spectrometry, serum organic and amino acids) was found to be normal. A chest computed tomographic (CT) scan showed ground-glass opacification and interlobular septal thickening (crazy paving pattern) (Fig. 2). Bronchoalveolar lavage (BAL) appeared milky; it was positive for periodic acid–Schiff (PAS) stain. A molecular genetics study for pulmonary surfactant metabolism dysfunction of seven genes (next-generation sequencing panel for *ABCA3*, *CSF2RA*, *CSF2RB*, *SFTPA1*, *SFTPB*, *SFTPC*, and *SFTPD* genes) showed no pathogenic mutation; however, a homozygous novel variant c.533G>A (p.Cys178Tyr) variant was detected in the *CSF2RA* gene. His serum granulocyte-macrophage colony-stimulating factor (GM-CSF) level was high (15.9 pg/ml, normal level <7.9 pg/ml) and GM-CSF autoantibodies were negative.

**Fig. 3:**
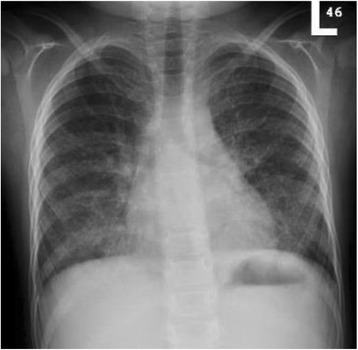
Chest X-ray 11 months after whole lung lavage with marked radiological improvement

Our case was diagnosed as having PAP that was likely to be congenital due to *CSF2RA* gene mutation so therapeutic WLL was performed. The procedure was performed under general anesthesia; he was intubated using an endotracheal tube of size 5.5 mm. Under fiberoptic bronchoscope (size 2.8 mm) direction, a bronchial blocker size 7 French was used to isolate his left lung. A bronchial blocker balloon was inflated and tested for stability and position under direct bronchoscopy observation; no leak was seen upon injecting saline through the catheter. After that, a whole left lung lavage was initiated using body temperature sterile normal saline. We injected 70 mL of normal saline each time, followed by chest physiotherapy and then aspiration. This procedure was repeated for his right lung. Each lung was washed with a total volume of 2 L and 40 mL. The left lung revealed 1968 mL back-aspirate and the right lung revealed 2001 mL as a total output. The fluid aspirate showed initially white milky fluid with time-dependent sedimentation. The fluid showed sequential progressive clearance toward the end of the procedure. He tolerated the procedure very well, apart from a transient drop in saturation to mid-80s, which responded very well to manual Ambu bagging; his fraction of inspired oxygen (FiO_2_) was kept at 1.0 throughout the procedure. Other than the transient drop in saturation, there was no major complication. He was transferred to our Pediatric Intensive Care Unit, and extubation was successful after 24 hours. After the first WLL his condition gradually showed improvement (respiratory rate was 30, saturated well on 0.5 to 1 L/minute nasal cannula oxygen 1 week after WLL), and after 11 months he became symptom-free with normal saturation on room air, with marked radiological improvement (Fig. 3).

Segregation analysis of the family showed that his parents are heterozygous and his 3.5-year-old sister is homozygous for the same mutation. Of interest, his sister is asymptomatic, with unremarkable physical examination findings (oxygen saturation 100% on room air) with no significant finding on chest X-ray. Currently, she is under regular follow-up and further investigation is postponed because she remains asymptomatic.

## Discussion

The surfactant is a mixture of proteins and lipids secreted by type II pneumocytes, cleared by alveolar macrophages under stimulation of GM-CSF [3]. In adults, the majority of cases of PAP cases autoimmune caused by autoantibodies directed against GM-CSF. Secondary PAP occurred due to impaired macrophage function from hematologic or solid malignancies, toxic dust inhalations, opportunistic infections, lysinuric protein intolerance, immunodeficiency, and immunosuppression agents [3, 4, 8]. In pediatric patients, most cases are of the congenital form and commonly occurred due to mutations in the genes involving surfactant production, that is *SFTPB*, *SFTPC*, *ABCA3*, and thyroid transcription factor 1 (*NKX2.1*) [1, 3, 4, 9, 10], or rarely due to mutations in the GM-CSF receptor (CSF2 α-chain receptor or CSF2 β-chain receptor) genes which is responsible for surfactant clearance [6, 7, 9]. Mutations in the genes involved in surfactant production cause PAP with interstitial lung disease, while mutations in the genes for surfactant clearance lead to pure PAP without involvement of interstitial space [11, 12].

The clinical presentation of congenital PAP due to *CSF2RA* defects varies from completely asymptomatic to severe symptoms with respiratory failure [5, 11]. The *CSF2RA* gene is found in the pseudoautosomal region 1 (PAR1) of the X and Y chromosomes; it is inherited in an autosomal recessive manner [13]. Diagnosis of PAP depends on clinical presentation in addition to the characteristic radiological findings on chest CT scan. Bronchoscopy usually confirms the diagnosis with the findings of typical milky BAL with positive PAS stain [1, 2]. The majority of cases of congenital PAP due to *CSF2RA* abnormalities developed progressive dyspnea, exercise intolerance, tachypnea, and hypoxemia at median age of 3.5 years with failure to thrive in 55% of patients [5]. The GM-CSF level is typically low in patients with autoimmune PAP and elevated in all patients with congenital PAP due to GM-CSF receptor abnormalities [11, 14].

Currently, the standard treatment for congenital PAP due to *CSF2RA* gene mutation is whole lung lavage [5]. All symptomatic patients who received whole lung lavage therapy experienced significant clinical improvement [5]. A single case report described a case with severe initial clinical presentation treated by bone marrow transplantation but the patient died due to respiratory infection 4 weeks after transplant [6]. Other therapeutic trials have been proposed such as pulmonary macrophage transplantation and gene therapy [15, 16].

In our case the patient had poor weight gain, progressive dyspnea since the age of 4.5 years with severe respiratory distress, typical radiological finding, his BAL was milky with positive PAS stain, and he had significant improvement after WLL. A novel variant of *CSF2RA* gene was detected in our case which has a typical clinical presentation for congenital PAP due *CSF2RA* defect, although this variant needs more work up to approve its pathogenicity.

His 3.5-year-old sister is asymptomatic despite the fact that she has homozygous variant but due to her relatively younger age she may develop symptoms later; as a result, further follow-up is warranted. Of note, clinical presentation of congenital PAP due to *CSF2R* defect can be triggered by factors such as respiratory tract infections [17]. Other potential explanations might be related to variation in penetrance, the involvement of modifier gene(s), environmental factors, or epigenetic changes [18].

## Conclusions

Our case has a typical presentation of congenital PAP due to *CSF2RA* defect, and we identified a novel variant in this gene likely to be pathogenic: c.533G>A (p.Cys178Tyr). Further studies are needed to confirm the pathogenicity of this variant and to characterize its clinical course further.

## Abbreviations

BAL: Bronchoalveolar lavage
*CSF2RA*: Colony stimulating factor 2 receptor alpha
CT: Computed tomographic
FiO_2_: Fraction of inspired oxygen
GM-CSF: Granulocyte-macrophage colony-stimulating factor
HIV: Human immunodeficiency virology
PAP: Pulmonary alveolar proteinosis
PAR1: Pseudoautosomal region 1
PAS: Periodic acid–Schiff stain
WLL: Whole lung lavage
